# Do home modifications reduce care home admissions for older people? A matched control evaluation of the Care & Repair Cymru service in Wales

**DOI:** 10.1093/ageing/afaa158

**Published:** 2020-09-18

**Authors:** Joe Hollinghurst, Richard Fry, Ashley Akbari, Alan Watkins, Neil Williams, Sarah Hillcoat-Nallétamby, Ronan A Lyons, Andrew Clegg, Sarah E Rodgers

**Affiliations:** Swansea University, Swansea, UK; Swansea University, Swansea, UK; Swansea University, Swansea, UK; Swansea University, Swansea, UK; Care & Repair Cymru, Cardiff, UK; Swansea University, Swansea, UK; Swansea University, Swansea, UK; University of Leeds, Leeds, UK; University of Liverpool, Liverpool, UK

**Keywords:** frailty, older people, care homes, interventions, administrative data

## Abstract

**Background:**

home advice and modification interventions aim to promote independent living for those living in the community, but quantitative evidence of their effectiveness is limited.

**Aim:**

assess the risk of care home admissions for people with different frailty levels receiving home advice and modification interventions against a control group who do not.

**Study design and setting:**

matched control evaluation using linked longitudinal data from the Secure Anonymised Information Linkage (SAIL) Databank, comprising people aged 60–95, registered with a SAIL contributing general practice. The intervention group received the Care & Repair Cymru (C & RC) service, a home advice and modification service available to residents in Wales.

**Methods:**

frailty, age and gender were used in propensity score matching to assess the Hazard Ratio (HR) of care home admissions within a 1-, 3- and 5-year period for the intervention group (*N* = 93,863) compared to a matched control group (*N* = 93,863). Kaplan–Meier curves were used to investigate time to a care home admission.

**Results:**

the intervention group had an increased risk of a care home admission at 1-, 3- and 5-years [HR (95%CI)] for those classified as fit [1-year: 2.02 (1.73, 2.36), 3-years: 1.87 (1.72, 2.04), 5-years: 1.99 (1.86, 2.13)] and mildly frail [1-year: 1.25 (1.09, 1.42), 3-years: 1.25 (1.17, 1.34), 5-years: 1.30 (1.23, 1.38)], but a reduced risk of care home admission for moderately [1-year: 0.66 (0.58, 0.75), 3-years: 0.75 (0.70, 0.80), 5-years: 0.83 (0.78, 0.88)] and severely frail individuals [1-year: 0.44 (0.37, 0.54), 3-years: 0.54 (0.49, 0.60), 5-years: 0.60(0.55, 0.66)].

**Conclusions:**

HRs indicated that the C & RC service helped to prevent care home admissions for moderately and severely frail individuals. The HRs generally increased with follow-up duration.

## Key points

- The likelihood of a care home admission has been shown to increase as frailty severity increases.
- Care & Repair Cymru provide home advice and modifications for people to live safely and independently at home.
- Routinely collected data can be used to help evaluate interventions for large cohorts.

## Introduction

### Background

Healthy ageing, maintenance of independence and reduced requirement for services are key challenges for policy makers, planners, commissioners and providers seeking to ensure sustainability of health and social care services internationally [1–3]. Projections of future care needs for older people in England and Wales indicate considerable challenges in this area, with an anticipated increase of 25% by 2025 [4]. These projections have major financial implications from an individual and societal perspective, with costs associated with residential and nursing home care particularly notable. In 2016, it was estimated that a self-funder would pay £44,000 per year for residential care, and local authorities would pay £32,300 per person per year [5].

Although care homes can help to provide essential care for those in need, older people typically prefer to remain living safely and independently in their own home wherever possible [6], or to relocate voluntarily for downsizing and convenience reasons [7]. Home-adaptations and advice are often provided to try to make homes safer for an individual and reduce the risk of adverse events such as premature care home admissions, but evidence of their effectiveness is limited [8]. A contributory factor is that primary research studies to evaluate interventions to prevent or delay transitions between independent living and care home residence are highly complex to implement, and resource intensive.

The use of existing anonymised routinely collected longitudinal data can help to provide large-scale data for studies on the effectiveness of interventions and provide robust evidence for commissioning decisions and policy [9]. In this study, we utilise the Secure Anonymised Information Linkage (SAIL) Databank [10–12] to investigate the effect of services provided by Care & Repair Cymru (C & RC) on the risk of care home admissions.

## Methods

### Data sources

Our cohort was created using data held within the SAIL Databank [10–12]. The SAIL Databank contains only anonymised records, and the anonymisation is performed by a trusted third party, the NHS Wales Informatics Service. The SAIL Databank has a unique individual anonymised person identifier known as an anonymous linking field (ALF) and unique address anonymised identifier known as a residential ALF (RALF) [13] that are used to link between data sources at individual and residential levels, respectively. Individual linking fields, nested within residences are both contained in the anonymised version of the Welsh Demographic Service dataset (WDSD), replacing the identifiable names and addresses of people who are registered with a free-to-use general practitioner (GP) service. The data held within SAIL are updated regularly, but the data are provisioned retrospectively on a per project basis. This project had data provisioned spanning from 1 January 2000 to 31 December 2017. The data contain records at the individual level, and all records have an associated date.

### Study design

We used longitudinal anonymised electronic health records (EHRs) and administrative data from the SAIL Databank to create a matched cohort study.

### Setting

Individuals in Wales aged 60–95 years who were registered with a GP submitting data to the SAIL Databank, this data are contained in the Welsh Longitudinal General Practice primary care data.

### Participants

We used primary care GP data collected from 1 January 2010 to 31 December 2017 to define our cohort (*N* = 553,765). SAIL currently contains approximately 80% of GP records for the population of Wales. GP data from 1 January 2000 to 31 January 2017 were used to define the level of frailty of individuals, by implementing the electronic frailty index (eFI) on a 10-year period prior to the index date [14,15]. We used intervention data from C & RC, mortality data from the Office of National Statistics (ONS) and demographic data from WDSD from 1 January 2010 to 31 December 2017 to capture care home admissions within 1-, 3- and 5-years of receiving an intervention. We included the 2014 Welsh Index of Multiple Deprivation (WIMD) quintiles in the descriptive data as a measure of socioeconomic status [16].

### Intervention group

C & RC is a national charitable body in Wales and actively works to ensure that older people have homes that are safe, secure and appropriate to their needs. We were able to anonymously upload and link the C & RC register to the SAIL Databank using a split file process. We used C & RC data to determine who had received a service from C & RC and when the work was completed based on the date supplied in the C & RC data. Our intervention cohort consisted of older people who received a C & RC service between 1 January 2010 and 31 December 2017 (*N* = 93,863). A list of the 100 most prevalent C & RC interventions and their counts are recorded in Appendix S1.

### Control group

Our control cohort was created by randomly assigning a service date from people receiving a C & RC service to those who did not. A matched control cohort was created based on a propensity score, calculated using logistic regression, with the following baseline variables: age, gender and eFI. We created a match (1:1 ratio) based on the nearest propensity score. Our sensitivity analyses repeated this method but increased the match ratio from 1:1 to 1:2 and 1:4 to increase the number of controls.

### Electronic frailty index

The eFI is based on an internationally established cumulative deficit model, and assigns a frailty score to an individual calculated using 36 variables from primary care GP data including symptoms, signs, diseases, disabilities and abnormal laboratory values, referred to as deficits [17]. The eFI score is the number of deficits present, expressed as an equally weighted proportion of the total. An individual with a single deficit would be assigned an eFI of 1/36 (0.03); another with nine deficits would be assigned an eFI of 9/36 (0.25). The eFI score is used to categorise individuals as: fit (eFI value of 0–0.12), mild (>0.12–0.24), moderate (>0.24–0.36) or severely frail (>0.36) [15]. We calculated the eFI retrospectively in the SAIL databank on the date a C & RC service was received (or randomly assigned comparator date) using 10 years of previous GP data for each individual. This meant that for someone receiving a C & RC service on 1 January 2010 the eFI was calculated using data from 1 January 2000 to 1 January 2010.

### Outcome of interest—care home admissions

The outcome of interest was a move to a care home. To determine the outcome, we created an index for anonymised care home addresses in the WDSD. The WDSD contains details of address changes declared to the NHS for the population of Wales. We therefore determined the date of a care home move by anonymously observing changes in residence for everyone in our cohort into any of the residences indexed as a care home in the WDSD. An anonymised care home index was created by using the Care Inspectorate Wales (CIW) [18] data source and assigning a Unique Property Reference Number (UPRN) to each address [19]. The UPRN was double-encrypted into a project level RALF and uploaded into SAIL to create a deterministic match to the WDSD.

**Table 1:** Cohort characteristics for the Care & Repair clients and the matched cohort with a 1:1 matching ratio

	Participants aged 60–95	C & RC clients	Matched non-clients (1:1)
*N*	553,765	93,863	93,863
Mean (SD) age	71.68 (8.50)	77.82 (8.34)	77.83 (8.35)
Female	296,883 (54%)	58,818 (63%)	58,874 (63%)
Male	256,882 (46%)	35,045 (37%)	34,989 (37%)
Frailty			
Fit	307,203 (55%)	28,457 (30%)	28,457 (30%)
Mild	173,715 (31%)	37,475 (40%)	37,492 (40%)
Moderate	59,050 (11%)	21,707 (23%)	21,791 (23%)
Severe	13,797 (2%)	6,224 (7%)	6,123 (7%)
WIMD 2014			
Least deprived 1	124,049 (22%)	18,765 (20%)	20,533 (22%)
2	109,768 (20%)	17,987 (19%)	18,066 (19%)
3	119,016 (21%)	20,196 (22%)	20,437 (22%)
4	105,312 (19%)	19,852 (21%)	17,641 (19%)
Most deprived 5	95,620 (17%)	17,063 (18%)	17,186 (18%)

## Statistical methods

### Cox regression

Cox regression was used to determine unadjusted and adjusted 1-, 3- and 5-year hazard ratios (HR), with 95% confidence intervals for the risk of a care home admission for the intervention group compared to the control group, people who did not receive a C & RC service. Adjusted HR included the propensity score as a covariate. We also stratified the results by eFI status to examine the effect of frailty.

### Kaplan–Meier

The Kaplan-Meier survival function was estimated for the 5-year period after a C & RC service was received (randomly assigned date for the control group). The results presented include stratification for C & RC client status and eFI category.

### Censoring

We censored our results if an individual did not have an event in the 5-year time period, died, or moved out of Wales any time prior to a care home admission.

## Results

### Descriptive data

The cohort characteristics as a result of the propensity score matching (matched on age, frailty and gender) are shown in Table 1. Appendix S2 shows the characteristics for the increased matching ratios used in sensitivity analyses. The number of care home admissions for the C & RC cohort and the 1:1 ratio matched cohort are shown in Appendix S3.

### Cox regression

The Cox regression results are presented in Table 2. The adjusted HRs had the propensity score included in the model. The results show a small increased hazard for C & RC clients as the time is increased from 1 to 5 years. When stratifying the data by frailty category we see that, irrespective of the length of time, C & RC clients defined as fit and mildly frailty have an increased HR for care home admission compared to non-clients, whereas the moderate and severely frail individuals have a reduced HR.

**Table 2:** Unadjusted and adjusted HR for care home admissions. The HRs were adjusted for the propensity score. The baseline (control) groups were people not receiving a C & RC intervention

		Stratified results			
	HR (95% confidence interval) for care home admissions for C & RC clients	Fit	Mild	Moderate	Severe
Adjusted HRs					
1 year	0.97 (0.91,1.04)	2.02 (1.73,2.36)	1.25 (1.09,1.42)	0.66 (0.58,0.75)	0.44 (0.37,0.54)
3 years	1.03 (0.99,1.07)	1.87 (1.72,2.04)	1.25 (1.17,1.34)	0.75 (0.70,0.80)	0.54 (0.49,0.60)
5 years	1.11 (1.08,1.15)	1.99 (1.86,2.13)	1.30 (1.23,1.38)	0.83 (0.78,0.88)	0.60 (0.55,0.66)
Unadjusted HRs					
1 year	0.98 (0.92,1.06)	2.01 (1.72,2.35)	1.25 (1.10,1.43)	0.66 (0.58,0.75)	0.45 (0.37,0.54)
3 years	1.06 (1.02,1.10)	1.86 (1.71,2.03)	1.28 (1.19,1.37)	0.77 (0.71,0.82)	0.56 (0.51,0.62)
5 years	1.15 (1.12,1.19)	1.97 (1.83,2.11)	1.34 (1.27,1.42)	0.86 (0.81,0.91)	0.64 (0.59,0.70)

### Kaplan–Meier

**Figure 1:**
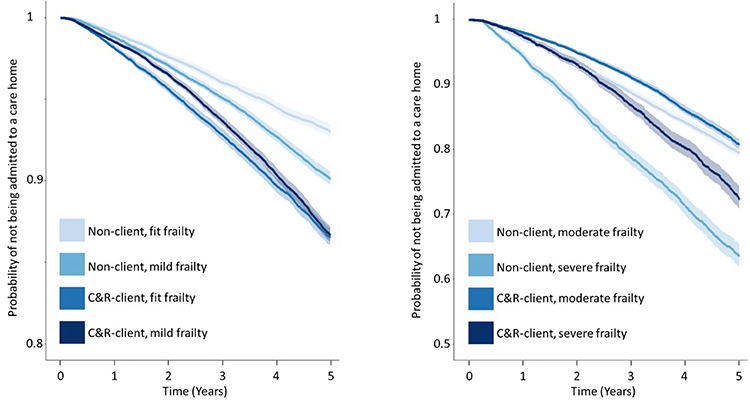
Kaplan–Meier survival curves for the probability of not moving to a care home, stratified by C & R status and eFI category: (a) The Kaplan–Meier curves for individuals with frailty statuses defined as fit and mild, (1) the probability scale is defined on the interval from 0.8 to 1. (b) The Kaplan–Meier curves for individuals with frailty statuses defined as moderate and severe, (2) the probability scale is defined on the interval from 0.5 to 1.

The Kaplan–Meier curves (Figure 1) show the probability of not moving to a care home over a 5-year period. The curves are stratified by the C & RC client status and eFI category.

### Sensitivity analyses

To test the sensitivity of the propensity score matching, we performed further matches with an increased match ratio. The cohort characteristics for the 1:2 and 1:4 match ratios are displayed in Appendix S2. The regression results are displayed in Appendix S4; the HRs generally agree with the results in Table 2. The sensitivity analyses also highlight the dependence on the Cox models to adjust the results for the characteristics included in the propensity scores. This can be seen by the increased difference between the adjusted and unadjusted HRs as the match ratio is increased. Specifically, as the cohort characteristics become more uneven, the adjustment has a larger effect.

## Discussion

Overall, the Cox regression results showed a small increase or statistically insignificant HR for C & RC clients. Stratifying by frailty status revealed differences that the non-stratified model failed to uncover. Other studies have shown the HR for a care home admission increased with frailty severity [14,15]. In this study, we have shown that the frailest C & RC clients have the biggest reduction in HR, indicating that C & RC prevent care home admissions for moderately and severely frail individuals. The HRs are generally lowest for those receiving the intervention in the 1- and 3-year time periods; indicating C & RC services benefit the clients most in these time frames.

The Kaplan–Meier curves showed that C & RC clients defined as fit and mildly frail had similar probability trajectories for care home admission. If care home admittance is an indication of frailty, this may suggest a misclassification of the eFI for the individuals in these categories. Specifically, frail individuals may present or self-refer themselves to C & RC rather than seeking support from a GP. This would mean a GP would not have the opportunity to record conditions that would increase an individual’s level of frailty defined by the eFI.

## Limitations

Although we were able to create large cohorts for our analyses, limitations include reliance on GP coding deficits and the use of routinely collected data. The demographic characteristics used within the propensity score matching (age, gender and eFI) show differences between the C & RC cohort and the general population (Table 1) and an increased difference as the match ratio is increased (Appendix S2). This highlights the difference in the characteristics of the C & RC clients compared to the potential controls in our data. Specifically, there are a higher proportion of females who are C & RC clients, and the C & RC clients are older and have a higher level of frailty. This shows that C & RC are potentially identifying the people at a high-risk of care home admission, and that careful consideration of a control group in the analysis is needed due to a potential health-need bias.

Currently, care home residency within SAIL is derived based on the CIW data source. This is different to other countries, where flags are created within administrative data to indicate care home residency [20]. Ideally, high-risk populations such as these would be accurately classified at the point of data capture. Concerns over poor recording in administrative data have been highlighted in previous studies [21,22]. This has led to the development of methods to combat poor coding of care home residency [23, 24].

## Conclusions

Organisations such as C & RC aim to promote independence in older people, so that people can live safely in their own homes. We found that C & RC helped to reduce the risk of care home admission over 1-, 3- and 5-year time periods for moderately and severely frail individuals, but there was an increased risk of moving to a care home for fit and mildly frail individuals. We interpret this as a combination of C & RC successfully identifying the people most in need of an intervention, and a potential misclassification of frailty using the GP data for the C & RC group.

Further work should be to communicate the importance of recording read codes for the identification of at-risk individuals with GP. This will lead to improved classifications and the potential for personalised care packages. There are also opportunities to cross check between primary care and other datasets such as secondary care outpatients and hospital admissions to systematically record data in order to reliably create detailed classifications of frailty.

## Supplementary Material

aa-19-1147-File002_afaa158
