# Supplementary material for: Do home modifications reduce care home admissions for older people? A matched control evaluation of the Care & Repair Cymru service in Wales
Source: Age Ageing. 2020 Sep 18;49(6):1056–61. doi: 10.1093/ageing/afaa158 (PMC7583515; doi:10.1093/ageing/afaa158)
Supplement: aa-19-1147-File002_afaa158 [file aa-19-1147-file002_afaa158.docx]

Do home modifications reduce care home admissions for older people? A matched control evaluation of the Care & Repair Cymru service in Wales

**SUPPLEMENTARY DATA**

- **Appendix 1.** Care & Repair 100 most prevalent interventions.
- **Appendix 2.** Propensity score matched comparators for the increased match ratios used in the sensitivity analyses
- **Appendix 3.** Numbers of care home admissions in 1, 3 and 5 year time periods for the total population and subgroups that were stratified by frailty status
- **Appendix 4.** Adjusted and unadjusted hazard ratios for care home moves in 1,3-and5-year time periods for 1:2 and 1:4 matching ratios.

Appendix 1. Care & Repair 100 most prevalent interventions.

| Work type | Count | Work type | Count |
| --- | --- | --- | --- |
| Grab Rails | 21,425 | Floor Coverings | 502 |
| Stair Rail | 11,417 | Gates | 485 |
| External Rails | 11,042 | Floor to ceiling pole | 480 |
| Other | 10,449 | Heating Repairs | 474 |
| Key Safes | 10,357 | Curtain Rails | 470 |
| Fire Safety Check | 7,270 | Boiler repairs | 461 |
| Telecare Equipment | 6,277 | Replace Boiler | 441 |
| Hand Rails | 6,216 | Half Step | 440 |
| Steps | 5,629 | Level Threshold | 438 |
| 12" Grab Rail | 4,063 | Fencing | 435 |
| Smoke Detector | 3,724 | Toilet Frames | 430 |
| Level Access Shower | 3,148 | Bathroom Repairs | 420 |
| Shower Seats | 2,686 | Medisafe | 416 |
| 18" Grab Rail | 2,511 | Shower screens | 368 |
| Bannister | 2,369 | Decorating | 364 |
| Stairlift | 2,185 | Door handle | 364 |
| CO Detector | 2,006 | Doorbell | 361 |
| Taps | 1,998 | Toilet replacement | 319 |
| Ramps | 1,983 | Floor Levelling | 315 |
| Newel Rails | 1,794 | Kitchen Repairs | 304 |
| Drop down rail | 1,770 | Bathroom Redesign | 303 |
| Keysafe Installation | 1,717 | Roof Repairs (full) | 285 |
| Lightbulbs | 1,585 | Rendering | 278 |
| Toilet repairs | 1,528 | Stairlift repair | 266 |
| Security Works | 1,440 | Brickwork Repairs | 265 |
| Gardening | 1,428 | Chimney Repairs | 254 |
| Plumbing | 1,360 | Showerhead Repair | 245 |
| Locks + Lock Repairs | 1,357 | Door Chains | 230 |
| Paths | 1,168 | Door Planning | 225 |
| Lever Taps | 1,145 | Telephone Socket | 215 |
| Roof Repairs (part) | 1,112 | Outside Lighting | 213 |
| Door - rehanging | 1,083 | Draught Proofing | 212 |
| Carpentry | 975 | Plastering | 204 |
| Moving Furniture | 959 | Electrical Rewire (full) | 203 |
| Lighting - additional | 951 | Loft Insulation | 188 |
| Window Repairs | 913 | Heat detector | 181 |
| cranked rail | 806 | Gutters - repairing | 177 |
| Shower alterations | 793 | Ceiling Repairs / Replacement | 176 |
| Door Replacement | 744 | Outbuilding Repairs | 175 |
| Exterior Lighting | 731 | Fascias & Soffits | 175 |
| 24" Grab Rail | 717 | Washing Line | 168 |
| Door Entry System | 646 | W.C. (Additional Facility) | 167 |
| Electrical Rewire (part) | 637 | Shower Tray | 163 |
| Bed/Chair Raisers | 636 | Electric Heating | 160 |
| Electrical Sockets | 586 | Blinds | 154 |
| Central Heating | 573 | Extension Single Storey | 145 |
| Gutters- clearing | 568 | Rainwater Goods | 139 |
| Leaks | 535 | Joint Visit | 135 |
| Damp/Condensation | 534 | Doors - widen | 132 |
| Window Replacement | 520 | Drainage Works | 132 |

Appendix 2. Propensity score matched comparators for the increased match ratios used in the sensitivity analyses

|  | C&R Clients | non-clients (1:1) | non-clients (1:2) | non-clients (1:4) |
| --- | --- | --- | --- | --- |
| N | 93,863 | 93,863 | 188,898 | 379,786 |
| Mean (SD) age | 77.82 (8.34) | 77.83 (8.35) | 76.58 (7.82) | 72.19 (7.64) |
| female | 58,818 (63%) | 58,874 (63%) | 114,597 (61%) | 213,981 (56%) |
| male | 35,045 (37%) | 34,989 (37%) | 74,301 (39%) | 165,805 (44%) |
| Frailty |  |  |  |  |
| Fit | 28,457 (30%) | 28,457 (30%) | 63,809 (34%) | 198,630 (52%) |
| Mild | 37,475 (40%) | 37,492 (40%) | 82,659 (44%) | 136,240 (36%) |
| Moderate | 21,707 (23%) | 21,791 (23%) | 34,860 (18%) | 37,343 (10%) |
| Severe | 6,224 (7%) | 6,123 (7%) | 7,570 (4%) | 7,573 (2%) |
| WIMD 2014 |  |  |  |  |
| Least deprived 1 | 18,765 (20%) | 20,533 (22%) | 41,966 (22%) | 86,404 (23%) |
| 2 | 17,987 (19%) | 18,066 (19%) | 36,666 (19%) | 75,351 (20%) |
| 3 | 20,196 (22%) | 20,437 (22%) | 40,874 (22%) | 81,650 (21%) |
| 4 | 19,852 (21%) | 17,641 (19%) | 35,449 (19%) | 70,799 (19%) |
| Most deprived 5 | 17,063 (18%) | 17,186 (18%) | 33,943 (18%) | 65,582 (17%) |

Appendix 3. Numbers of care home admissions in 1, 3 and 5 year time periods for the total population and subgroups that were stratified by frailty status

|  | 1 year | | 3 years | | 5 years | |
| --- | --- | --- | --- | --- | --- | --- |
|  | C&R clients | Non-clients | C&R clients | Non-clients | C&R clients | Non-clients |
| Non-movers | 92,362 | 92,308 | 88,717 | 88,820 | 85,613 | 86,391 |
| Care home admissions | 1,501 | 1,555 | 5,146 | 5,043 | 8,250 | 7,472 |
| Stratification |  |  |  |  |  |  |
| Frailty - Fit | C&R clients | Non-clients | C&R clients | Non-clients | C&R clients | Non-clients |
| Non-movers | 27,986 | 28,218 | 26,947 | 27,616 | 26,125 | 27,225 |
| Care home admissions | 471 | 239 | 1,510 | 841 | 2,332 | 1,232 |
| Frailty - Mild | C&R clients | Non-clients | C&R clients | Non-clients | C&R clients | Non-clients |
| Non-movers | 36,975 | 37,083 | 35,754 | 36,086 | 34,637 | 35,258 |
| Care home admissions | 500 | 409 | 1,721 | 1,406 | 2,838 | 2,234 |
| Frailty - Moderate | C&R clients | Non-clients | C&R clients | Non-clients | C&R clients | Non-clients |
| Non-movers | 21,320 | 21,197 | 20,347 | 19,958 | 19,492 | 19,102 |
| Care home admissions | 387 | 594 | 1,360 | 1,833 | 2,215 | 2,689 |
| Frailty - Severe | C&R clients | Non-clients | C&R clients | Non-clients | C&R clients | Non-clients |
| Non-movers | 6,081 | 5,810 | 5,669 | 5,160 | 5,359 | 4,806 |
| Care home admissions | 143 | 313 | 555 | 963 | 865 | 1,317 |

Appendix 4. Adjusted and unadjusted hazard ratios for care home moves in 1,3-and5-year time periods for 1:2 and 1:4 matching ratios.

|  | Adjusted HR 1:2 match ratio (lower 95% CI , upper 95% CI) | | | | Unadjusted HR 1:2 match ratio (lower 95% CI , upper 95% CI) | | |  |  |
| --- | --- | --- | --- | --- | --- | --- | --- | --- | --- |
|  | 1 year | 3 years | 5 years | 1 year | | 3 years | 5 years | | |
| C&R Clients | 1.04  (0.97,1.11) | 1.10  (1.06,1.14) | 1.18  (1.15,1.22) | 1.34  (1.26,1.43) | | 1.46  (1.41,1.51) | 1.58  (1.54,1.63) | | |
| *Frailty* | | | | | | | | |  |
| Fit | 2.02  (1.78,2.29) | 1.99  (1.85,2.13) | 2.06  (1.95,2.18) | 2.15  (1.90,2.44) | | 2.12  (1.97,2.27) | 2.17  (2.05,2.30) | | |
| Mild | 1.34  (1.19,1.51) | 1.32  (1.24,1.41) | 1.38  (1.31,1.45) | 1.76  (1.57,1.98) | | 1.78  (1.67,1.89) | 1.86  (1.77,1.95) | | |
| Moderate | 0.67  (0.59,0.76) | 0.76  (0.71,0.81) | 0.84  (0.80,0.89) | 0.82  (0.73,0.93) | | 0.96  (0.90,1.03) | 1.10  (1.04,1.16) | | |
| Severe | 0.44  (0.36,0.53) | 0.54  (0.49,0.60) | 0.60  (0.56,0.66) | 0.45  (0.37,0.55) | | 0.58  (0.52,0.64) | 0.66  (0.61,0.72) | | |
|  | *Adjusted HR 1:4 match ratio (lower 95% CI , upper 95% CI)* | | | *Unadjusted HR 1:4 match ratio (lower 95% CI , upper 95% CI)* | | | | | |
|  | 1 year | 3 years | 5 years | 1 year | | 3 years | 5 years | | |
| C&R Clients | 1.16  (1.09,1.24) | 1.22  (1.18,1.27) | 1.32  (1.29,1.37) | 2.54  (2.38,2.71) | | 2.79  (2.69,2.89) | 3.05  (2.97,3.14) | | |
| *Frailty* |  |  |  |  | |  |  | | |
| Fit | 2.51  (2.22,2.85) | 2.42  (2.26,2.59) | 2.50  (2.37,2.65) | 5.56  (4.93,6.27) | | 5.41  (5.07,5.78) | 5.46  (5.18,5.76) | | |
| Mild | 1.40  (1.24,1.58) | 1.38  (1.30,1.47) | 1.45  (1.38,1.52) | 2.71  (2.42,3.04) | | 2.78  (2.62,2.96) | 2.95  (2.81,3.09) | | |
| Moderate | 0.68  (0.60,0.76) | 0.76  (0.71,0.82) | 0.85  (0.80,0.89) | 0.88  (0.78,1.00) | | 1.03  (0.97,1.10) | 1.18  (1.12,1.24) | | |
| Severe | 0.44  (0.36,0.53) | 0.54  (0.49,0.60) | 0.60 (0.56,0.66) | 0.45  (0.37,0.55) | | 0.58  (0.52,0.64) | 0.66  (0.61,0.72) | | |
